# Bias modelling in evidence synthesis

**DOI:** 10.1111/j.1467-985X.2008.00547.x

**Published:** 2009-01

**Authors:** Rebecca M Turner, David J Spiegelhalter, Gordon C S Smith, Simon G Thompson

**Affiliations:** Medical Research Council Biostatistics UnitCambridge, UK; University of CambridgeUK; Medical Research Council Biostatistics UnitCambridge, UK

**Keywords:** Bias, Elicitation, Evidence synthesis, Heterogeneity, Meta-analysis

## Abstract

Policy decisions often require synthesis of evidence from multiple sources, and the source studies typically vary in rigour and in relevance to the target question. We present simple methods of allowing for differences in rigour (or lack of internal bias) and relevance (or lack of external bias) in evidence synthesis. The methods are developed in the context of reanalysing a UK National Institute for Clinical Excellence technology appraisal in antenatal care, which includes eight comparative studies. Many were historically controlled, only one was a randomized trial and doses, populations and outcomes varied between studies and differed from the target UK setting. Using elicited opinion, we construct prior distributions to represent the biases in each study and perform a bias-adjusted meta-analysis. Adjustment had the effect of shifting the combined estimate away from the null by approximately 10%, and the variance of the combined estimate was almost tripled. Our generic bias modelling approach allows decisions to be based on all available evidence, with less rigorous or less relevant studies downweighted by using computationally simple methods.

## 1. Introduction

Standard methods for meta-analysis assume that all studies have addressed the same question in a similar way and therefore provide information on common parameters of interest. In practice, however, the studies vary in degree of rigour and in relevance to the research question. Rigour (or lack of internal bias) reflects how well a study estimates its intended parameters and varies according to use of randomization, adequacy of allocation concealment, degree of blinding and attrition levels. Relevance (or lack of external bias) is defined with respect to a target question. For example, in a typical comparison of two interventions, the objective is to compare the effect of the interventions on a specified primary outcome in a particular population. The source studies that are available may differ from this target setting, with respect to study population, outcomes measured and details of interventions. This paper is motivated primarily by meta-analyses that were carried out to inform policy decisions, where a target question is predefined, but varying relevance also affects the interpretation of any meta-analysis.

Differences in rigour and relevance between studies which are potentially eligible in a systematic review are typically addressed by choosing a minimum threshold for inclusion. This introduces an arbitrary division and valuable evidence may be discarded (Gluud, 2006). Of additional concern is a failure to adjust for the remaining internal and external biases in the studies selected. The majority of systematic reviews assess the methodological quality of their source studies (de Craen *et al.*, 2005), but only about 50% make use of the quality assessment and this is usually only through sensitivity analyses or exploratory subgroup analyses (Moja *et al.*, 2005). In general, the primary analysis of the review makes no allowance for differences in rigour and relevance. Consequently, biases in the studies included are incorporated in the overall results.

If a bias affecting a particular study were of known magnitude, bias adjustment could be achieved simply by shifting the study effect estimate and interval. Typically, however, the internal and external biases are of unknown magnitude, and this uncertainty should be acknowledged in the analysis, resulting in a wider interval as well as a shift of location. It is misleading to report confidence intervals (CIs) which reflect only the extent of possible random error when systematic biases are suspected (Greenland, 2005). The uncertainty which is due to systematic biases is, unfortunately, more difficult to quantify and cannot be evaluated by using statistical methods alone. It has been recommended that scientific judgement is used to evaluate this uncertainty, using all the relevant information that is available (Taylor and Kuyatt, 1994).

Several methods of adjusting for biases in meta-analysis have been proposed. Weighting the analysis by quality scores is known to be inadequate (Greenland and O’Rourke, 2001); among other reasons, many quality items contributing to the score may have no association with bias, and there is no allowance for the direction of biases. Spiegelhalter and Best (2003) acknowledged potential biases in a set of studies by assuming an additive bias in the effect of interest (treatment effect, for example). Distributions that are chosen directly for parameters representing the internal and external bias in each study influence the relative weighting of studies in the pooled analysis. Other researchers have adjusted for multiple sources of bias by using a model-based approach: distributions are specified for a set of parameters which together determine the bias in the target effect, and separate parametric models are used for each specific source of bias. Eddy *et al.* (1992), in their confidence profile method for meta-analysis, presented models for multiple internal and external biases and incorporated these into the likelihood to obtain bias-adjusted results. Wolpert and Mengersen (2004) similarly used a full likelihood approach to adjust for biases due to eligibility violation, and misclassification of exposure or outcome. Greenland (2005) constructed separate models for the bias due to uncontrolled confounding, non-response and misclassification of exposure and used Monte Carlo simulation methods and approximate adjustments to sample and adjust for the individual biases, rather than taking a full likelihood approach.

Our objective in this paper is to provide simple generic methods for allowing for internal and external biases in meta-analysis and evidence synthesis. The term ‘evidence synthesis’ refers to a broader class of analyses, encompassing synthesis of results from diverse sources, e.g. different study designs, different treatment comparisons and different clinical end points (Ades and Sutton, 2006). Our approach to bias adjustment is intermediate in complexity between the multiple-bias modelling approaches that were discussed above and the simpler approach that was taken by Spiegelhalter and Best. We model biases due to individual sources, as did Eddy *et al.* (1992), Wolpert and Mengersen (2004) and Greenland (2005), but assume a direct form for the bias in the target parameter, as did Spiegelhalter and Best (2003). Estimation of bias-adjusted results is achieved by using moment-based methods, and we provide tools for elicitation of distributions for the biases in a set of studies. We aim to present methods which are accessible in a routine analysis setting.

We develop our methods in the context of an example in antenatal care, which concerns the effectiveness of routine anti-D prophylaxis in pregnant women who are Rhesus negative. The purpose of anti-D prophylaxis is to prevent ‘sensitization’ of the mother, to avoid haemolytic disease of the newborn in subsequent pregnancies. A technology appraisal (Chilcott *et al.*, 2003) that was commissioned by the UK National Institute for Clinical Excellence (NICE) identified 11 relevant studies, which were subject to many internal and external biases. Many of the studies were historically controlled, only one was a randomized trial and doses, populations and outcomes varied between studies and differed from the target UK setting. The principal conclusions in the NICE appraisal were based on meta-analysis of the two studies that were considered most relevant, which were selected according to dose and population type.

The outline of this paper is as follows. Section 2 discusses identification of internal and external biases. In Section 3, we present our approach to adjustment for biases which may be assumed to act additively on the intervention effect, and in Section 4 our tools for quantifying bias. Section 5 reports the application of these methods to the anti-D prophylaxis example. In Section 6, we describe methods for adjusting for biases which depend on the magnitude of the intervention effect. Section 7 discusses incorporation of empirical evidence on bias.

## 2. Identifying internal and external biases

To make allowance for internal and external biases in an evidence synthesis, we must first identify all potential sources of bias in the set of selected studies. It is helpful to break this task down into a series of smaller steps. The first step is to write down a precise definition of the target question, which should describe the population to whom the results of the evidence synthesis will be generalized, details of the target comparison (e.g. intervention and control policies, in a standard two-arm comparison) and the outcome of interest.

When identifying the biases that are present in a study, it is not trivial to distinguish external from internal biases. For example, consider a study which recruited different populations in the intervention and control arms, neither identical to the target population. It is clear that differences between populations will cause both internal and external bias with respect to the target question, but unclear how to determine the extent of each bias. To address this problem, we first envisage an idealized version of each source study and write down a miniprotocol for it. The idealized study can be viewed as a repeat of the original study by using a design which eliminates all sources of internal bias. In the miniprotocol, we specify the population which the researchers planned to study, their planned comparison and the outcome which they planned to measure. This information is usually easily extracted from the abstract and early sections of the study publication. In the idealized study, the population recruited and the outcome measured would be identical in the intervention and control arms. It may be helpful to think of the idealized study as a perfect randomized controlled trial, but note that this design need not be practicable, for instance where ethical issues prevent use of randomization or blinding.

**Fig. 1:**
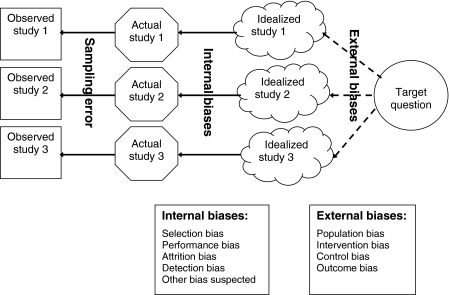
Identifying internal and external biases

The process of identifying internal and external biases is now much simpler (Fig. 1). To identify internal biases, we compare the details of the completed source study against the miniprotocol for its idealized study. To identify external biases, we compare each idealized study against the target question. An additional advantage of separating the two tasks is that they can be undertaken independently by different members of the research team. For example, in the medical field, it may be desirable that internal biases are assessed by a group that is led by a statistician or epidemiologist, and external biases by a group that is led by a clinician.

To reiterate our approach proposed above, the steps are as follows.

- Define the target question.
- Write down a miniprotocol for an idealized version of each study.
- Identify internal biases by comparing the original study against the idealized study.
- Identify external biases by comparing the idealized study against the target question.

Tools for completion of steps (c) and (d) are provided in Section 4.

For bias modelling, we would like to group individual biases into broader categories of bias, which may be assumed to be independent (see Fig. 1). For internal bias, we have chosen to use the following categorization which has been used elsewhere (Higgins and Green, 2005; Deeks *et al.*, 2003): selection bias; performance bias; attrition bias; detection bias. Selection bias refers to systematic differences between comparison groups at baseline. Performance bias represents systematic differences other than the study comparison; this may be caused by inadequate blinding of participants in trials and by misclassification of exposure in observational studies. Attrition bias reflects systematic differences between comparison groups in exclusions and drop-outs. Detection bias describes systematic differences between groups in outcome assessment, which may result from a lack of standardization and blinding of those assessing the outcome. We have added a further internal bias category, ‘other bias suspected’, to represent the opinion that additional biases seem likely. For example, this would cover suspicion that there are inconsistencies in a published study which suggest erroneous results.

For external bias, the bias categories represent the differences between the idealized studies and the four components of the target question: population bias; intervention bias; control bias; outcome bias. For example, population bias may reflect differences in age, sex or health status of the idealized study participants, compared with the target population. Intervention bias refers to differences in dose, timing or delivery method of the idealized study intervention compared with the target intervention, and similarly control bias refers to differences in the control strategy. When the target question is a comparison of two active interventions, intervention bias and control bias would be relabelled appropriately. Outcome bias may represent differences in the definition, timing or method of measurement of the idealized study outcome compared with the target.

There may be some flexibility over how to define the idealized study, e.g. where a study has measured several outcomes and none is identical to the target outcome. The aim is to maximize the information that is supplied by the study with respect to the target question and this could mean weighing up relevance against the extent of missing data. We do not here address publication bias or selective reporting of outcomes, for which methods have been discussed elsewhere (Rothstein *et al.*, 2005; Williamson and Gamble, 2005). These issues should be confronted before considering potential biases within the data set that is available.

## Simple approach to bias adjustment

In this section, we propose a simple method of adjusting for internal and external biases in meta-analysis. We wish to estimate some underlying quantity *θ*, which is the ‘true’ intervention effect in our target setting. For a two-arm comparison, *θ* represents the difference in the target end point between the target intervention and control policies, when assessed within the target population. The intervention effect is defined on a scale on which it is reasonable to assume that most biases act additively.

Suppose that there are *k* studies, *i*=1,…,*k*, and each provides a statistic *y*_*i*_ (with sampling variance 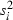 assumed known) as a conventional estimator of some underlying study-specific quantity *θ*_*i*_, which is the true intervention effect according to the idealized protocol for study *i*. We assume that, if there were no *internal* biases, then for each study 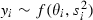, where the notation *f*(*μ*,*σ*^2^) represents a general distribution with mean *μ* and variance *σ*^2^. We assume also that, if there were no *external* biases, all the studies would be measuring the target quantity so *θ*_*i*_=*θ*. Thus internal biases reflect that the above sampling distribution is not a correct assumption, whereas external biases reflect that *θ*_*i*_ is not equal to *θ*. All the development below carries through by using moments and does not require assumptions of normality.

The method of adjustment that is proposed here is appropriate only for biases which may be regarded as independent of the magnitude of the intervention effect, and we refer to these as additive biases. Examples are given in Section 4.1, and in Section 6 we discuss methods of adjusting for biases which are proportional to the intervention effect.

### 3.1. Incorporating internal biases

We use superscripts I and E to distinguish internal and external biases. For multiple independent sources *j*=1,…,*J*^I^ of internal bias, we write 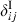 to denote the effect of bias source *j* on the estimated intervention effect in study *i*. 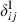 is unknown, and we assume that our uncertainty about 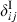 is represented by a distribution 
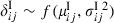
. Methods for elicitation of 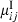 and 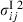 are presented in Section 4. Having elicited 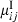 and 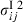, assuming independence between sources of bias means that the total internal bias in the *i*th study is 
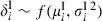
, where 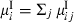 and 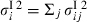.

To allow for internal biases, we assume an additive bias model 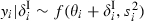, or equivalently 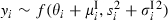
(1) or equivalently 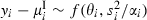, where 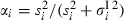. *α*_*i*_ can be interpreted as a quality weight for rigour, representing the proportion of within-study variability that is unrelated to internal biases (Spiegelhalter and Best, 2003). Highly rigorous studies would have *α*_*i*_ near to 1, whereas less rigorous studies would have lower *α*_*i*_ and would thus be downweighted in the meta-analysis.

### 3.2. Incorporating external biases

We assume multiple independent sources *j*=1,…,*J*^E^ of external bias and write 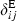 to denote the effect of bias source *j* in study *i*. As for the internal biases, a distribution 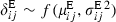 is assumed for each bias in each study. We include an additional variance parameter *τ*^2^ to represent unexplained between-study heterogeneity. If adjusting perfectly for internal and external biases, there would be no remaining heterogeneity, so the estimate of *τ*^2^ provides a gauge of our success in addressing the biases. Having elicited 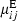 and 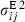, the total external bias in the *i*th study is 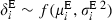, where 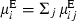 and 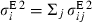. Then the external bias model may be written as 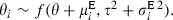


We note that this is not a standard random-effects model for meta-analysis, which would assume that all *θ*_*i*_ are ‘exchangeable’, i.e. have the same distribution.

Thus, assuming no internal biases, we have 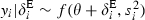, or equivalently 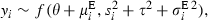
(2) or equivalently, when *τ*^2^ is non-zero, 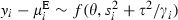, where 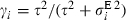 can be interpreted as a relevance weight, representing the proportion of between-study variability that is unrelated to external biases (Spiegelhalter and Best, 2003). Idealized studies which are highly relevant to the target setting would have *γ*_*i*_ near to 1, whereas less relevant studies with lower *γ*_*i*_ would be downweighted in the random-effects analysis.

### 3.3. Adjusting for both internal and external biases

By combining the earlier models (1) and (2), we obtain a model which incorporates internal and external biases, 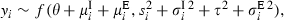
(3) or equivalently 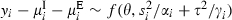. This can be interpreted as a random-effects meta-analysis formulation, in which the location is adjusted for systematic biases, and within- and between-study variability are multiplied by quality weights for rigour and relevance respectively.

The inverse variance estimator of *θ* is 
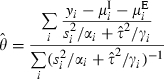


with approximate standard error 
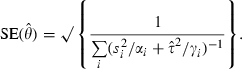


Assuming approximate normality for the estimator 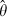, a 95% CI for *θ* is 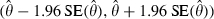. This assumption is based on a central limit theorem argument, noting again that we do not require normality in the individual bias distributions.

To perform these calculations, an estimate for *τ*^2^ is required and it is convenient to use a method-of-moments estimate that is based on the heterogeneity statistic *Q* (DerSimonian and Laird, 1986). Here, this is derived from a fixed effect estimate which allows for internal and external biases, 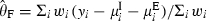, where 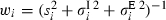. The heterogeneity statistic is given by 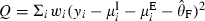 and a moment estimate for *τ*^2^ is 
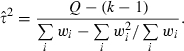
(4)

The estimate is set to 0 if 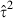 is negative. In the setting of meta-analysis, negative values would represent greater variation within than between studies, which is not considered plausible. The relevance weights *γ*_*i*_ are now calculated by using the estimate 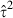.

## 4. Quantifying bias

To adjust for internal and external biases by using the moment-based method that was presented in Section 3, we require distributions for each bias in each study. These would ideally be based on empirical evidence. However, although some empirical studies of bias have been published, these report estimates and CIs for average bias only and do not provide measures of between-study variation in bias. We return to this and give a brief review of the relevant literature in Section 7. In the absence of empirical evidence, we propose a two-stage approach for constructing the required bias distributions from elicited opinion. First, checklists are used to extract qualitative details of study characteristics which could potentially cause bias, for each of the five internal and four external bias categories. Next, distributions for each bias are quantified, through marking estimated ranges on elicitation scales.

### 4.1. Checklists for bias

The items that are included in the checklists for sources of internal and external bias (Tables 1 and 2) are based on the Downs and Black (1998) checklist for assessing the methodological quality of randomized and non-randomized studies. The checklists are completed with reference to the original study publications, the target question and miniprotocols for idealized versions of each study (as discussed in Section 2). To complete the checklist for internal bias (Table 1), we compare the details that are provided by the study publication against the idealized study. Answers should be given with regard to the results for the chosen idealized outcome, since potential for bias may vary between outcomes within a study. Study features which led to each answer are described briefly in the ‘Description’ column of Table 1.

**Table 1:** Checklist for sources of internal bias†

*Question*	*Yes/no/unclear*	*Description*
*Selection bias*		
Subjects in different intervention groups recruited from same populations?		
Subjects in different intervention groups recruited over same time periods?		
Inclusion and exclusion criteria clear?		
Randomization used?		
Adequate allocation concealment?		
Comparable baseline characteristics?		
Adequate adjustment for confounding? [In observational studies only]		
Does extent of *selection bias* depend on magnitude of intervention effect?		
*Performance bias*		
Subjects blinded?		
Care givers blinded?		
Did subjects receive their assigned (or reputedly received) interventions?		
Does extent of *performance bias* depend on magnitude of intervention effect?		
*Attrition bias*		
Are the results unlikely to be affected by losses to follow-up?		
Are the results unlikely to be affected by *post hoc* exclusions?		
Does extent of *attrition bias* depend on magnitude of intervention effect?		
*Detection bias*		
Outcome assessors blinded?		
Outcome measured accurately?		
If the results were back-calculated from a reported analysis, was the statistical analysis appropriate?		
Does extent of *detection bias* depend on magnitude of intervention effect?		
*Other bias suspected*		
Does extent of *other bias* depend on magnitude of intervention effect?		

†The empty areas are to be completed.

The bias checklist includes a judgement of whether each bias depends on the magnitude of the intervention effect. We treat biases which do not as ‘additive’ biases, and biases which do as ‘proportional’ biases, and our elicitation process and model for adjustment differ according to type. For example, the external bias that is caused by a study's dose differing from the target dose of treatment is proportional to the intervention effect, whereas the internal bias that is caused by recruiting two study groups from different populations is independent of the intervention effect. Proportional biases are addressed in Section 6. The assumption that all biases can be categorized as either additive or proportional is a simplification, which we return to in Section 8.

To complete the checklist for external bias (Table 2), we compare the details of each idealized study against the target question. As in Table 1, a description of relevant study characteristics is recorded alongside the answer to each question and the checklist includes a judgement of whether each bias depends on the intervention effect. With an adequate ‘idealized protocol’, assessment of external biases does not require access to the original study publication and could (or even should) therefore be carried out without knowledge of the study results. It is also desirable that internal biases are assessed blind, but the practicalities are more difficult. In principle, the results in the study publications could be concealed by a third party so that internal biases are assessed without knowledge of the study results. However, this does not overcome the problem that assessors who work in a closely related field may already know the study results. Assessors should certainly not base their opinions concerning size of additive biases on the magnitude of observed effect or sampling variance, since this would violate the assumption of independence between the additive biases and the effect estimate *y*_*i*_.

**Table 2:** Checklist for sources of external bias†

*Question*	*Yes/no/unclear*	*Description*
*Population bias*		
Study subjects in idealized study drawn from population identical to target population, with respect to age, sex, health status etc.?		
Does extent of *population bias* depend on magnitude of intervention effect?		
*Intervention bias*		
Active intervention in idealized study identical to target active intervention in dose, timing etc.?		
Does extent of *intervention bias* depend on magnitude of intervention effect?		
*Control bias*		
Control intervention in idealized study identical to target control intervention?		
Does extent of *control bias* depend on magnitude of intervention effect?		
*Outcome bias*		
Study outcome for idealized study identical to target outcome?		
Does extent of *outcome bias* depend on magnitude of intervention effect?		

†The empty areas are to be completed.

### 4.2. Elicitation scales

The completed bias checklists (Tables 1 and 2) are used to inform an opinion on the size of each bias affecting each study. In this section, we discuss elicitation for all additive biases that are identified in Tables 1 and 2, which do not depend on the magnitude of the intervention effect. We return to proportional biases in Section 6. Below we consider elicitation of the biases affecting relative risk, but the methods could be easily modified for other measures of intervention effect.

Ideally, several assessors would be asked to provide their opinion on the size of each bias in every study. For each internal bias category, the assessor is asked to consider the following question, taking into account the information that is extracted in Table 1.

Even if there were no intervention effect in this study, what apparent effect (ignoring sampling variation) might be induced by this bias?

**Fig. 2:**
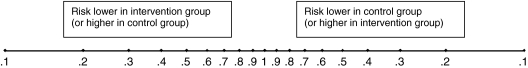
Elicitation scale for quantifying additive bias

The assessor is given a copy of an elicitation scale (Fig. 2) for each bias and asked to mark a 67% range for the relative risk of the adverse outcome chosen in that study such that they feel that the true answer to the question is twice as likely to lie inside rather than outside this range. Our decision to elicit an interval corresponding to moderate rather than high probability is based on findings that peoples’ performance at assessing intervals tends to be better for lower levels of certainty (O’Hagan *et al.*, 2006).

The relative risk scale that is shown is symmetric with respect to the study arms, so a doubling of risk in the control group compared with the intervention group is marked as 0.5 on the left-hand half of the scale, whereas a doubling of risk in the intervention group is marked as 0.5 on the right-hand half. Range limits marked symmetrically around 1 represent a belief that the bias is equally likely to be in favour of the control as in favour of the intervention. Relative risk values are marked on a log-scale, since this will be used when constructing probability distributions.

**Fig. 3:**
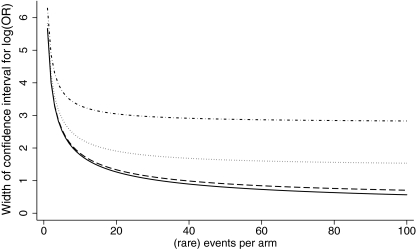
Effect of ranges for bias on the approximate width of the CI for the bias-adjusted log-odds-ratio, assuming rare events and no intervention effect (ranges refer to a symmetric relative risk scale, as shown in Fig. 2): 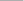, no bias; 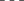, 67% range (0.9, 0.9); 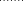, 67% range (0.7, 0.7); 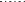 67% range (0.5, 0.5)

To ease the process of choosing numerical limits for each bias, we recommend that assessors first make a qualitative judgement of the severity of bias, before quantifying their opinion as a 67% range. Assessors write down their judgement of the severity of bias (as none, low, medium or high) in favour of the intervention and, separately, the severity of bias in favour of the control. We suggest the following correspondence between qualitative judgements of severity and choices for the range limit: none (1); low (0.9–1); medium (0.7–0.9); high (less than 0.7). These divisions are guided by consideration of the effect of different ranges of bias on the CI for a log-odds-ratio (Fig. 3). For convenience, we assume that events are rare and that the intervention has no effect. In a trial which observes 10 events per arm, adjustment for a bias with 67% range (0.9, 0.9) has little effect; the standard error for the log-odds-ratio is increased by only 2.7%, which is equivalent to reducing the number of observed events to 9.5 per arm. Adjustment for a bias with a wider range of (0.7, 0.7) or (0.5, 0.5) causes the standard error to increase by 28% or 84% respectively, which is equivalent to reducing the number of events to 6.1 or 2.9 per arm.

For each external bias category, the assessor is asked to consider the following question, taking into account the information that is extracted in Table 2.

Even if there were no effect in a study addressing the target question, what effect might there be in an idealized version of the present study (i.e. assuming no internal biases)?

For each external bias in every study, the assessor is asked to mark a 67% range on an elicitation scale (Fig. 2), as described above for internal bias.

To construct a probability distribution from the range estimate that is chosen for each bias, we work on the log-scale. For bias *j* in study *i*, the range limits *a*_*ij*_ on the left-hand half of the elicitation scale and *b*_*ij*_ on the right-hand half correspond to range limits log (*a*_*ij*_), log (1/*b*_*ij*_) on a scale representing the log-relative-risk for intervention compared with control. For convenience, we assume that the 67% range represents approximately *μ*_*ij*_±*σ*_*ij*_, as it would under normality, and obtain the following elicited values for *μ*_*ij*_ and *σ*_*ij*_: 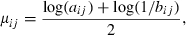

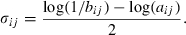


In this way, parameters *μ*_*ij*_ and 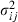 that are required for application of the methods that were proposed in Section 3 are elicited from each assessor for all categories of additive bias in every study. The bias checklists and elicitation scales that are used may be obtained from a technical report that is available on line at http://rsb.info.nih.gov/ij/download.html)

## 5. Example

### 5.1. Effectiveness of routine anti-D prophylaxis

Our example concerns the antenatal care of women who are Rhesus negative, i.e. women who have no D antigen on their red blood cells. When a Rhesus negative woman is pregnant with a Rhesus positive fetus, there is a danger of ‘sensitization’, where contact between fetal blood and maternal blood causes the mother to produce antibodies to the D antigens in the fetal red blood cells (Contreras, 1998). In any future pregnancies with a Rhesus positive fetus, these antibodies may cross the placenta and lead to a condition that is known as haemolytic disease of the newborn, which can in severe cases result in stillbirth, disability or neonatal death. Under the preventative policy that was introduced in many countries in 1969, Rhesus negative mothers are offered a dose of a blood product called anti-D immunoglobulin after birth of a Rhesus positive baby. Women are also offered anti-D immunoglobulin after miscarriage or abortion, and over time this has been extended to include other potentially sensitizing events such as amniocentesis (which is an invasive procedure in which a sample of the amniotic fluid surrounding the fetus is extracted for testing) and occurrences of vaginal bleeding. Under this policy, the frequency of haemolytic disease of the newborn is dramatically reduced, but cases still occur in about 1 in 21000 births. In 2001, the NICE in the UK commissioned an evaluation of the clinical effectiveness and cost effectiveness of additionally offering routine antenatal anti-D immunoglobulin to all unsensitized Rhesus negative women.

The NICE technology appraisal (Chilcott *et al.*, 2003) identified 11 relevant studies. Since the methodological quality of these was generally poor, the study results were potentially affected by many internal biases. In addition, the dose of anti-D immunoglobulin that was used, the obstetric history of the women who were studied and the timing of outcome measurement varied between studies and often differed from the target UK setting, causing multiple external biases. Owing to these problems, the final NICE guidance was based principally on a meta-analysis of only the two studies that were considered most relevant (Mayne *et al.*, 1997; MacKenzie *et al.*, 1999), both of which evaluated the effectiveness of offering the target dose of anti-D immunoglobulin to *primigravidae* (women who are pregnant for the first time) in the UK. This analysis provided strong evidence in favour of routine antenatal injection of anti-D immunoglobulin, with the odds ratio of sensitization estimated as 0.37 (95% CI 0.21, 0.65). The policy proposed was also considered to be cost effective and, in 2002, the NICE recommended that it should be implemented across the UK.

### 5.2. Quantifying biases in the anti-D immunoglobulin studies

We would like to quantify the internal and external biases in the anti-D immunoglobulin studies, to perform a bias-adjusted meta-analysis. This would address the concerns over internal biases and varying relevance, while avoiding the need for rather arbitrary selection of studies. We begin by using the tools that were presented in Section 4 to elicit probability distributions for all additive biases. For convenience and for comparability with the NICE appraisal results, we estimate the study treatment effects on the log-odds-ratio scale. The odds ratio may be assumed to approximate the relative risk since the outcome is rare.

The advantages of eliciting judgements from several assessors rather than a single assessor are clear, and we weighed up the benefits of

- individual elicitation followed by mathematical pooling *versus*
- group elicitation, where a single consensus distribution is reached through discussion.

Our chosen elicitation process draws on both approaches.

- One assessor completes checklists for sources of bias (Tables 1 and 2).
- All assessors read the original papers alongside the bias checklists and meet to discuss queries and to reach a consensus on completed checklists.
- Each assessor marks ranges on elicitation scales (Fig. 2), independently of other assessors.
- Distributions from different assessors are pooled mathematically.

In this way, we hope to gain the advantages of group elicitation, knowledge sharing and resolution of misunderstandings, without the disadvantage that one individual could overly influence the choice of consensus distribution (O’Hagan *et al.*, 2006). As an additional safeguard against simple errors, assessors are given feedback on exceptional instances of extremely divergent opinion and are allowed to correct their opinion if a mistake has been made.

To demonstrate the methods, we discuss in detail the additive biases that were identified in one of the anti-D immunoglobulin studies (Hermann *et al.*, 1984), before proceeding to a bias-adjusted meta-analysis. Hermann *et al.* (1984) reported sensitization status at 8 months *postpartum* (after delivery) for two groups of Rhesus negative women who were delivered of Rhesus positive babies in a Swedish hospital (Table 3). Women in the intervention group received 1250 international units (IUs) of anti-D immunoglobulin at about 32–34 weeks of gestation during the period 1973–1977, and comparison was made against other women who had been studied over the period 1968–1977 in earlier unpublished research. The target question for the NICE appraisal of anti-D immunoglobulin is shown in Table 4, together with a miniprotocol for an idealized version of Hermann *et al.* (1984), and we use these to identify the internal and external biases as discussed in Section 2.

**Table 4:** Parameters of NICE target question and idealized version of Hermann *et al.* (1984)

	*Target question*	*Idealized version of Hermann et al. (1984)*
Population	Unsensitized pregnant Rhesus negative women in the UK	Unsensitized Rhesus negative women delivered of Rhesus positive babies at the Växjö hospital in Sweden
Intervention	Dose of 500 IUs anti-D immunoglobulin offered at 28 and 34 weeks’ gestation, in addition to control antenatal care	Dose of 1250 IUs anti-D immunoglobulin given at 32–34 weeks’ gestation, in addition to control antenatal care
Control	Anti-D immunoglobulin offered *postpartum* and after potentially sensitizing events during pregnancy	Anti-D immunoglobulin offered *postpartum* and after potentially sensitizing events during pregnancy
Outcome	Rhesus sensitization which will affect a subsequent pregnancy	Rhesus sensitization at 8 months *postpartum*

**Table 3:** Studies of the effectiveness of anti-D prophylaxis for prevention of postnatal sensitization in Rhesus negative women

*Study*	*Design*	*Population receiving anti-D prophylaxis*	*Dose*	*Outcome*	*Sensitizations/total*		*Log-odds-ratio*	*Variance of log-odds-ratio*
							
					*Treated*	*Control*		
Bowman *et al.* (1978)	Non-randomized, historical controls	Unsensitized pregnant Rhesus negative women	1500 IUs at 28 and 34 weeks	Sensitization during pregnancy or within 3 days of delivery	0/1357	62/3533	−3.89	2.02
Hermann *et al.* (1984)	Non-randomized, historical controls	Unsensitized pregnant Rhesus negative women	1250 IUs at 32–34 weeks	Sensitization at 8 months *postpartum*	2/529	10/645	−1.42	0.60
Huchet *et al.* (1987)	Quasi-randomized study (allocation based on year of birth)	Pregnant Rhesus negative *primiparae*†	500 IUs at 28 and 34 weeks	Sensitization at 2–12 months *postpartum*	1/472	7/468	−1.97	1.15
Lee and Rawlinson (1995)	Randomized trial	Rhesus negative *primigravidae*‡	250 IUs at 28 and 34 weeks	Sensitization at 6 months *postpartum*	3/361	6/405	−0.58	0.51
MacKenzie *et al.* (1999)	Non-randomized, contemporary controls	Rhesus negative unsensitized *primiparae*†	500 IUs at 28 and 34 weeks	Sensitization in a subsequent pregnancy	12/3320	26/3146	−0.83	0.12
Mayne *et al.* (1997)	Non-randomized, historical controls	Rhesus negative *primigravidae*‡	500 IUs at 28 and 34 weeks	Sensitization in a subsequent pregnancy	4/1425	16/1426	−1.39	0.31
Tovey *et al.* (1983)	Non-randomized, historical controls	Rhesus negative *primigravidae*†	500 IUs at 28 and 34 weeks	Sensitization in a subsequent pregnancy	2/325	22/582	−1.85	0.55
Trolle (1989)	Non-randomized, historical controls	Unsensitized pregnant Rhesus negative women	1500 IUs at 28 weeks	Sensitization at 10 months *postpartum*	0/291	6/322	−2.48	2.16

† *Primipara*: a woman who has given birth for the first time to an infant or infants, alive or stillborn (after 20 weeks’ gestation).

‡ *Primigravida*: a woman who is pregnant for the first time.

Using the bias checklists (Tables 1 and 2), we find that a major concern in this study is selection bias: the controls were recruited in an earlier time period than women receiving the intervention, no inclusion–exclusion criteria are presented for the control group and no attempt was made to adjust for confounding. A lack of blinding means that performance bias is likely: subjects (or care givers) may differ by group in willingness to report (or treat with anti-D immunoglobulin) potentially sensitizing events such as bleeding. Attrition seems probable though not reported, in that the available denominators represent the number of women who were delivered of Rhesus positive babies, but the outcome is measured 8 months later. Additionally, there are post-baseline exclusions from the intervention group. There are also minor issues over the lack of blinding of outcome assessors (detection bias), a difference from the target population (population bias) and a difference from the target control strategy (control bias). Biases related to dose and timing of anti-D immunoglobulin are deferred to Section 6.

**Fig. 4:**
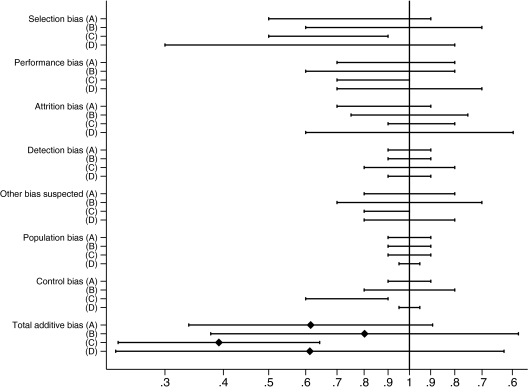
Additive biases in Hermann *et al.* (1984), 67% ranges of distributions elicited from four assessors (A–D) and means and 67% ranges for total additive bias

Fig. 4 shows the distributions that were elicited for additive biases in Hermann *et al.* (1984) from four different assessors (the authors of this paper). Values on the left-hand side of the axis represent bias which decreases the risk in the intervention group compared with the control group, i.e. which would make the intervention appear more effective. Several of the biases in Hermann *et al.* (1984) are expected to favour the intervention, though uncertainty is high and many of the distributions that were elicited have a wide spread in both directions. The assessors’ opinions on the biases vary, but not dramatically so. All four distributions for total additive bias are wide, with an expected value which favours the intervention.

**Fig. 5:**
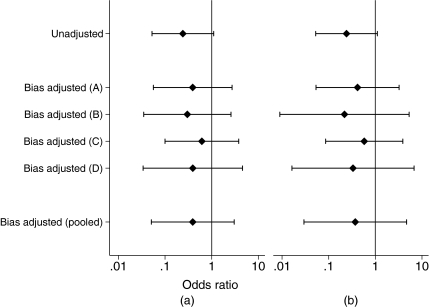
Effect of adjusting for (a) additive bias and (b) all bias on the odds ratio (with 95% CIs) in Hermann *et al.* (1984)

The effect of adjusting the study results for total additive bias is illustrated in Fig. 5(a), for the four assessors separately and pooled. We decided to pool across assessors after summing the biases rather than before, because individuals may differently categorize potential sources of bias. The pooled distribution for the bias-adjusted result is based on medians of the means and standard deviations of the four assessors’ distributions for the bias-adjusted result. In principle, we could choose to give more weight to certain assessors’ opinions on the basis of their greater knowledge in particular areas, but selection of such weights would be difficult in practice.

The effect of adjustment on the odds ratio and its 95% CI is not as great as might be expected when looking at the bias distributions in Fig. 4, because the estimate from the Hermann *et al.* (1984) study is fairly imprecise anyway (Table 5). The unadjusted odds ratio for sensitization, comparing women receiving routine antenatal anti-D immunoglobulin against controls, is 0.24 (95% CI 0.05, 1.10). After allowance for additive biases under the assumptions of model (3), the odds ratio is adjusted to 0.39 (95% CI 0.05, 3.04). The estimate has shifted towards 1 and, rather than bordering on statistical significance, the upper limit of the CI now extends well beyond the null value.

**Table 5:** Summary of results for Hermann *et al.* (1984) and for meta-analysis of eight anti-D immunoglobulin studies, before and after adjusting for biases: estimated odds ratio of sensitization, comparing routine anti-D immunoglobulin against control, and corresponding ‘effective number of events’ in each arm†

*Bias adjustment*	*Results for Hermann et al. (1984) analysis*			*Results for meta-analysis*			
	*Odds ratio (95% CI)*	*Effective number of events*		*Odds ratio (95% CI)*	*Effective number of events*		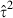
		*Treated*	*Control*		*Treated*	*Control*	
None	0.24 (0.05, 1.10)	2.1	8.5	0.28 (0.17, 0.46)	19.7	70.8	0.05
Additive biases	0.39 (0.05, 3.04)	1.3	3.3	0.29 (0.14, 0.57)	10.4	36.1	00
All biases	0.37 (0.03, 4.68)	0.8	2.2	0.25 (0.11, 0.56)	7.0	28.5	00

† Details are given in Section 5.2.

To help those viewing the results to interpret the effect of bias adjustment, we convert the odds ratio results into numbers of events that would be observed in each arm of a study of rare events. Failure to allow for bias can be viewed as similar to overcounting rare events. In a large study with similar sample sizes in each arm, the estimated log-odds-ratio 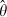 and its variance 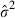 are approximately equal to log (*r*_1_/*r*_0_) and 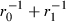 respectively, where *r*_0_ and *r*_1_ represent the number of observed events in the control and treated arms. For a given 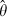 and 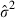, we can therefore calculate the ‘effective number of events’ as 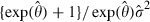 in the control arm and 
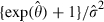
 in the treated arm. In Hermann *et al.* (1984), adjustment for additive biases is found to be analogous to removing more than half of the total effective number of events (Table 5).

### 5.3. Bias-adjusted meta-analysis

Our original aim in the anti-D immunoglobulin example was to perform a bias-adjusted meta-analysis of the 11 relevant studies that were identified by the NICE appraisal (Chilcott *et al.*, 2003). However, we have decided to exclude one study which made comparison with a published control rate, for which no denominator was available (Parsons *et al.*, 1998). In addition, three of the 11 studies used the same group of control women. The second and third of these added in results for women who had received the intervention since completion of the first study, without recruiting further controls (Bowman and Pollock, 1978, 1987). We consider only the first of these as a genuine comparative study (Bowman *et al.*, 1978) and exclude the other two. The example now comprises eight comparative studies of the effectiveness of routine antenatal injection of anti-D immunoglobulin in the prevention of sensitization of pregnant Rhesus negative women (Bowman *et al.*, 1978; Hermann *et al.*, 1984; Huchet *et al.*, 1987; Lee and Rawlinson, 1995; MacKenzie *et al.*, 1999; Mayne *et al.*, 1997; Tovey *et al.*, 1983; Trolle, 1989). The observed results are shown in Table 3, together with brief details of the study designs. In a conventional random-effects meta-analysis of these data, the odds ratio of sensitization, comparing routine anti-D immunoglobulin treatment against control, is estimated as 0.28 (95% CI 0.17, 0.46). The between-study variance for the log-odds-ratio is estimated as 0.22^2^=0.05.

**Fig. 6:**
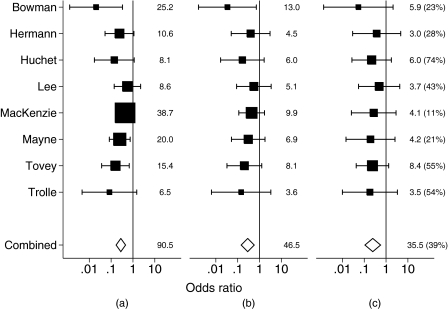
Meta-analysis of eight studies evaluating the effectiveness of routine anti-D prophylaxis—unadjusted and bias-adjusted odds ratios (with 95% CIs) (for each result, the corresponding total ‘effective number of events’ is listed alongside): (a) unadjusted; (b) bias adjusted (additive); (c) bias adjusted (all)

As described for the Hermann *et al.* (1984) study in Section 5.2, we elicited distributions for the additive biases in the other seven anti-D immunoglobulin studies. Under the assumptions of model (3), we adjust the study estimates and standard errors and perform a bias-adjusted meta-analysis. The unadjusted and additive bias-adjusted study results are shown in Figs 6(a) and 6(b), together with the unadjusted and additive bias-adjusted meta-analysis results. The majority of the estimates have shifted towards the null value and the standard errors for the intervention effect have increased for all studies. After adjustment for additive bias, the total effective number of events that is represented by the results has fallen substantially for most studies (Fig. 6). We note that the effective number of events represents both the magnitude and the precision of the treatment effect. For example, the effective number of events remains relatively high for Bowman *et al.* (1978), where the treatment effect is extreme, though imprecisely estimated. The effect of bias adjustment is greater for more precise studies. Among the anti-D immunoglobulin studies, the effect is most dramatic for MacKenzie *et al.* (1999), which was the largest study and gave very precise results before adjustment for bias. Here, the standard error almost doubles and the total effective number of events is quartered after bias adjustment.

In a bias-adjusted meta-analysis, the odds ratio of sensitization is estimated as 0.29 (95% CI 0.14, 0.57), and the between-study variance *τ*^2^ is estimated as 0. There remains strong evidence for the effectiveness of routine antenatal anti-D treatment, but the uncertainty has increased (Table 5). The effect of bias adjustment on the overall odds ratio is analogous to halving the total number of events in a large rare events study (Table 5).

Through adjusting for internal and external additive biases, we have reduced the estimated heterogeneity to 0 and appear to have explained the systematic differences between the studies. The reduction in heterogeneity is caused partly by a decrease in variability of the study estimates, and partly by an increase in within-study uncertainty. After adjustment for additive biases, the variance of the log-odds-ratio estimates has decreased by 27%, whereas a measure of the typical within-study variance (Higgins and Thompson, 2002) has approximately doubled.

## 6. Adjusting for proportional biases

### 6.1. Models for adjustment

We now consider biases which depend on the magnitude of the intervention effect. Examples include the external bias that is caused by a study delivering the intervention to only a subset of the target population, and the internal bias due to a study excluding non-compliers who did not receive the intervention. The first of these reduces the intervention effect in the idealized study compared with the target, whereas the second exaggerates the effect in the actual study compared with the idealized study. These biases cannot be modelled by using additive bias models, and we assume that they reduce or increase the intervention effect multiplicatively.

Assuming multiple independent sources *k*=1,…,*K*^I^ of internal proportional bias, we write 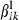 to represent the effect of an internal proportional bias source *k* on the estimated intervention effect in study *i*, and similarly we write 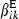 to represent an external bias. In the absence of additive biases, we would allow for a single internal proportional bias 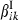 by assuming the model 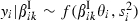. To allow for a single external proportional bias 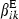 in the absence of additive biases or unexplained between-study heterogeneity, we would assume that 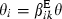.

The total internal proportional bias in study *i* is 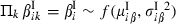, and similarly the total external proportional bias is 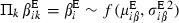. Methods for eliciting the proportional biases *β*_*ik*_ and deriving 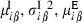 and 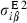 are discussed in Section 6.2. To allow for internal additive bias 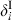 as well as internal proportional bias 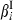, we assume the model 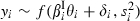. To allow for external additive bias 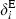 and proportional bias 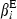, we assume that 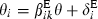. A model which incorporates internal and external biases is then 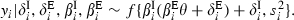


We recall the distributions that were assumed earlier for the additive biases 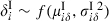 and 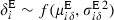, now written with a subscript *δ*. Using the distribution of the product of independent variables (Armitage and Berry, 2001), we obtain 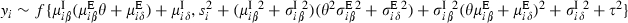
(5) where *τ*^2^ again represents unexplained between-study heterogeneity.

When using data from a single study *i* and a single set of elicited distributions for the additive and proportional biases in study *i*, the bias-adjusted estimate of the target intervention effect is 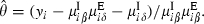
(6)

The formula for the corresponding standard error of 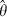 is a function of the intervention effect *θ*, for which we substitute the estimate 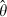: 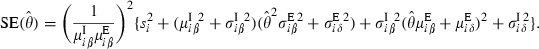
(7)

As when adjusting for additive biases, we consider it preferable to accumulate bias distributions within assessors before pooling across assessors. The effects of additive and proportional biases are combined during adjustment of each study's results. To obtain pooled bias-adjusted results for a particular study, we therefore adjust for each assessor separately as above, then average adjusted results across assessors. A bias-adjusted meta-analysis is performed by fitting a standard random-effects model to the adjusted effect estimates and their standard errors, again using a moment estimate for any unexplained heterogeneity *τ*^2^. Alternative approaches are discussed as sensitivity analyses in Section 6.4.

### 6.2. Elicitation of bias distributions

The information in the completed bias checklists (Tables 1 and 2) is used to inform opinion on the size of each proportional bias in each study. For each bias category identified as proportional, whether internal or external, the assessor is asked to consider the following question.

What proportional change to the intervention effect (represented by the log-relative-risk, ignoring sampling variation) might this bias induce?

**Fig. 7:**
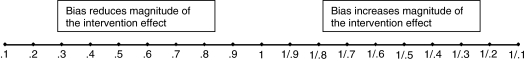
Elicitation scale for quantifying proportional bias

The assessor is given a copy of an elicitation scale (Fig. 7) for each bias and asked to mark the value which they feel is most likely, together with a 67% range for the reduction or exaggeration that is induced in the intervention effect, such that they feel that the true answer is twice as likely to lie inside rather than outside this range. The assessor is also asked to consider whether only exaggeration of the intervention effect is possible and reduction is impossible (or vice versa), and if so to indicate this by marking a bound at 1. The scale that is shown is symmetric with respect to the proportional reduction or exaggeration.

As for the additive biases, we fit symmetric distributions to the logarithms of the range estimates that were chosen for each proportional bias *k* in study *i*, assuming that the 67% range represents approximately *λ*_*ik*_±ω_*ik*_ on the log-scale. To find moments for total internal proportional bias and total external proportional bias, which are products of multiple proportional biases on the original scale, we assume log-normality (Evans *et al.*, 1993). Total internal proportional bias then has expectation 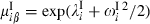 and variance 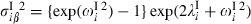, where 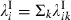 and 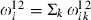, with similar expressions for total external proportional bias.

This approach does not acknowledge the bounds at 1 which assessors marked for some biases. As a sensitivity analysis in Section 6.4, we fit beta distributions to biases which were believed to be bounded while continuing to assume symmetric distributions for unbounded biases. The beta distribution is fitted to the mode and outer range limit, whereas the log-normal distribution that was described above is fitted to the two range limits. In future elicitations, we would prefer the visual scale in Fig. 7 to be logarithmic (as in Fig. 2), since this scale is used in analysis.

### 6.3. Quantifying proportional biases in the anti-D immunoglobulin studies

Intervention bias, the external bias that is caused by differences between the study intervention and the target intervention, is present in many of the anti-D immunoglobulin studies, and the size of this bias is plausibly proportional to the magnitude of the treatment effect. The interventions differ from the target in up to three ways: the dose of anti-D immunoglobulin that was used, timing of occasions given and delivery policy, which refers to the definition of the population receiving the intervention and whether the intervention was offered or given (i.e. those refusing not included).

In Hermann *et al.* (1984), the intervention differed from the target in dose and timing and in that only women who received the intervention were included in the study (Table 4). Another proportional bias arises in Hermann *et al.* (1984) as a detection bias, since Hermann *et al*. (1984) admitted that the cases of sensitization that were identified may have included false positive results. False positive results in both arms cause dilution of the intervention effect, and the resulting bias is greater when the intervention is more effective. Outcome bias arises in Hermann *et al.* (1984) through sensitization being measured at 8 months after delivery rather than during a subsequent pregnancy (Table 4). This may cause false positive and false negative results and may similarly be assumed proportional to the intervention effect.

**Fig. 8:**
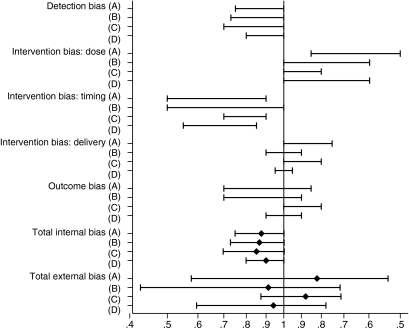
Proportional biases in Hermann *et al.* (1984), 67% ranges of distributions elicited from four assessors (A–D) and means and 67% ranges for total internal and external proportional bias

Fig. 8 shows the distributions that were elicited for proportional biases in Hermann *et al.* (1984) from the same four assessors, together with the resulting distributions for total internal and external proportional bias. Values below 1 represent a reduction in the magnitude of the intervention effect, whereas values above 1 represent an exaggeration. It was clear that the detection bias that is caused by false positive cases of sensitization would reduce the intervention effect, as would giving anti-D immunoglobulin on only one rather than two occasions, whereas the higher dose would exaggerate the effect in comparison with the target UK setting, though the assessors’ opinions differ somewhat on the sizes of these effects and the associated uncertainty. Opinions over the size and direction of the effects of the delivery policy and timing of outcome measurement in Hermann *et al.* (1984) are varied, but the 67% ranges that were elicited are overlapping.

The effect of adjusting the Hermann *et al.* (1984) study results for both proportional and additive bias is illustrated in Fig. 5(b), for the four assessors separately and pooled. The pooled adjusted estimate and standard error are obtained as medians of the four assessors’ adjusted estimates and standard errors. Adjusting for proportional bias causes a noticeable widening in the 95% CI for two of the assessors but, after pooling, the additional effect of adjusting for proportional bias is not very great. After allowance for all biases under the assumptions of model (5), the odds ratio is 0.37 (95% CI 0.03, 4.68). In comparison with the results that were adjusted for additive bias only, the estimate has moved slightly further from the null and the interval is wider (Table 5). The corresponding effective number of events in a large study of rare events falls by approximately a third in both arms (Table 5).

### 6.4. Full bias-adjusted meta-analysis

As described for the Hermann *et al.* (1984) study in Section 6.3, we elicited distributions for the proportional biases in the other seven anti-D immunoglobulin studies. Under the assumptions of model (5), we adjust the study estimates and standard errors and perform a meta-analysis that is adjusted for all additive and proportional biases. Fig. 6(c) presents the results from this analysis. In comparison with the results that were adjusted for additive bias only, the standard error increases for five of eight of the studies, and the magnitude of the change in standard error tends to be larger in these studies. Again, the most noticeable effect is seen for MacKenzie *et al.* (1999), where the standard error of the intervention effect increases by a ratio of 1.74 (comparing Figs 6(b) and 6(c)). Comparing against the unadjusted results, the overall effect of bias adjustment is highest for MacKenzie *et al.* (1999) and Mayne *et al.* (1997) (Fig. 6). We recall that the NICE conclusions were based principally on these two studies, which were chosen on the basis of relevance to the target UK setting (Chilcott *et al.*, 2003).

In a meta-analysis that is adjusted for additive and proportional bias, the odds ratio of sensitization is estimated as 0.25 (95% CI 0.11, 0.56). In comparison with the unadjusted results, the estimate has shifted from the null value by just over 10%, whereas the variance has almost tripled (Table 5). The effective number of events has fallen to 36% (treated arm) and 40% (control arm) of the numbers that are represented by the unadjusted meta-analysis result (Table 5). However, there remains strong evidence for the effectiveness of routine antenatal anti-D prophylaxis.

In the analysis that was adjusted for all biases, the between-study variance *τ*^2^ is again estimated as 0. In comparison with the unadjusted analysis, the variance of the log-odds-ratio estimates has decreased by 61% and the typical within-study variance (Higgins and Thompson, 2002) has almost tripled. In general, we expect adjustment for internal and external biases to remove much of the heterogeneity in a meta-analysis. If substantial heterogeneity remained, we would suspect a problem with the process of bias adjustment or a problem with combining the set of studies. For example, one or more sources of bias may not have been adequately addressed. To investigate, one could look carefully at the outlying studies, obtain a second opinion on the bias checklists for these and check for errors in the analysis.

To investigate the robustness of these results, we additionally perform the meta-analysis under several variations on the assumptions that were made above. The first of these acknowledges the bound at 1 which assessors declared for some proportional biases. A beta distribution is fitted to the mode and outer range limit, using a binary search algorithm since the beta distribution percentiles cannot be expressed in closed form. Distributions for total internal and external proportional bias are then not easily derived, since they may represent products of multiple beta and log-normal distributions, so we obtain these through forward simulation. The odds ratio estimate and CI from this meta-analysis are identical to two decimal places to those from the analysis ignoring the bounds.

In a second sensitivity analysis, we take a different approach to pooling across assessors. Pooled distributions for each study are obtained for each of total internal additive bias, total external additive bias, total internal proportional bias and total external proportional bias, based on medians of the four assessors’ means and standard deviations. The observed results from each study are adjusted for the pooled bias distributions by using equations (6) and (7), and then a standard random-effects meta-analysis is performed. This analysis gives an odds ratio of 0.26 (95% CI 0.11, 0.58), which is very close to the initial analysis. As a third variation on the analysis that was described in Section 6.1, we adjust the study-specific standard errors by using an estimate for 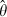 which is common across studies, for each assessor separately. Estimation of a common 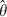 is achieved by taking an iterative approach to fitting a random-effects meta-analysis model, where the study variances are functions of 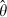. In this analysis, the odds ratio is estimated as 0.24 (95% CI 0.10, 0.54), which is again very close to the initial results.

**Fig. 9:**
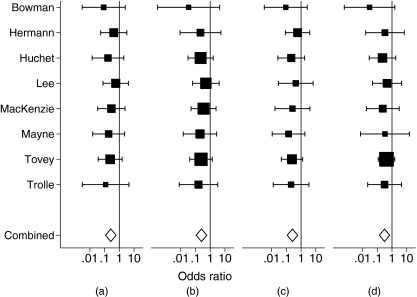
Meta-analysis of anti-D immunoglobulin studies adjusted for additive and proportional biases, using the elicited opinions of assessors (a) A, (b) B, (c) C and (d) D separately (with 95% CIs)

Finally, we perform four separate bias-adjusted meta-analyses by using the elicited bias distributions that were given by each assessor (Fig. 9). The bias-adjusted odds ratio is estimated as 0.26 (0.12, 0.60), 0.25 (0.11, 0.58), 0.26 (0.11, 0.59) and 0.31 (0.14, 0.70), when using the opinions that were elicited from assessors A, B, C and D respectively. The difference in results between assessor D and assessors A–C are largely caused by differing opinions over the biases in Tovey *et al.* (1983). In a bias-adjusted meta-analysis of the other seven anti-D immunoglobulin studies, the results that were based on each single assessor's opinions on biases are very similar across assessors. In all these sensitivity analyses, the between-study variance *τ*^2^ was estimated as 0.

In the anti-D immunoglobulin example, the bias-adjusted results seem robust to different methods of pooling across assessors and are little affected by whether the bounds are acknowledged when fitting distributions to proportional biases. The sensitivity analyses increase our confidence in the results that were obtained from the initial bias-adjusted meta-analysis. Programs for implementing the bias adjustment methods can be obtained by contacting the authors.

## 7. Incorporating empirical evidence on bias

Our method of bias adjustment requires distributions to be specified for each category of bias in every study. Currently, we have based all distributions on elicited opinion alone, but it would be desirable for the bias distributions to be informed by empirical evidence wherever possible. Empirical evidence on bias can be obtained from meta-epidemiological studies, which pool data from a collection of meta-analyses to study the effects of particular study characteristics. Many meta-epidemiological studies have investigated the effects of selection bias on the magnitude of treatment effect, through comparing randomized and non-randomized studies (Deeks *et al.*, 2003), or looking at the effect of inadequate allocation concealment and inadequate allocation sequence generation (Schulz *et al.*, 1995; Moher *et al.*, 1998; Kjaergard *et al.*, 2001; Juni *et al.*, 2001; Balk *et al.*, 2002). Some of the latter studies have also examined the effect on treatment effect of a double-blind study design and of reported attrition. Analysis of meta-epidemiological data sets could inform the assessors’ opinions during the elicitation process or could supply empirical distributions to be used directly for particular biases. To take either approach would require measures of between-study variation in bias, but the meta-epidemiological studies that have been carried out to date have only reported estimates and CIs for the average effects of bias.

A model for extracting empirical information on bias from a meta-epidemiological data set has been proposed recently (Welton *et al.*, 2008; Sterne *et al.*, 2008). This was exemplified in assessing the bias due to inadequate allocation concealment by using the data that were obtained by Schulz *et al.* (1995). The model that was constructed for the observed data distinguishes three components of variability in bias: uncertainty in mean bias; between-study (within-meta-analysis) variability in bias; between-meta-analysis variability in bias. In our elicitation approach to obtaining distributions for bias, we elicit the combined variance for each bias, which is the sum of these components. Elicitation alone could not provide estimates for these separate components of bias uncertainty. Welton *et al.* (2008) used the empirical evidence on bias to provide prior distributions for a model which combines results from studies of low and high quality (i.e. with inadequate and adequate allocation concealment). The new meta-analysis was assumed exchangeable with previous meta-analyses and the studies included were not assessed in detail. Under this model, the variance for a low quality study includes the estimated variance for mean bias and the between-meta-analysis variance in bias, which cannot be compensated for by increasing the number of low quality studies. If relying on generic meta-epidemiological data alone, a set of biased studies thus has a limit to the precision for estimating the target effect. In our approach, we gain information by assessing the biases in our particular studies rather than assuming exchangeability with previous meta-analyses.

Our objective in modelling biases is to make adjustment for all sources of internal and external bias. In this context, it would be extremely rare to be able to base all bias distributions on empirical evidence alone. Where there are external biases, these are specific to the differences between the source study and target question, and meta-epidemiological studies are unlikely to provide relevant information. For certain internal biases, empirical information from meta-epidemiological studies could inform the elicitation, and assessors could modify the generic bias distributions according to the severity of bias in each study.

## 8. Discussion

In this paper, we propose methods for adjusting for the varying rigour and relevance of studies that are included in a typical evidence synthesis. We have presented a strategy for identifying internal and external biases, tools for eliciting quantitative distributions for the biases and simple models for adjustment. Our objective is to describe an approach allowing decisions to be based on all available evidence, with less rigorous or less relevant studies discounted by using computationally simple methods.

Our aim was to find an approach that can be implemented in a routine analysis setting and this governed our choice of methods. A method-of-moments estimate was used for the excess heterogeneity *τ*^2^, though it may in principle be preferable to use an iterative method of estimation (DerSimonian and Kacker, 2007). Alternatively, the models could be fitted within a full Bayesian framework, making proper allowance for the uncertainty in *τ*^2^ and the study sampling variances 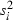 (Smith *et al.*, 1995). A full Bayesian analysis could also incorporate biases that are elicited through a flexible elicitation procedure (Oakley and O’Hagan, 2007). For simplicity, we have assumed log-normality throughout; we checked the results against those from fitting beta distributions to the proportional biases and found no difference in our example.

When pooling elicited distributions across multiple assessors, we take averages of the adjusted estimates and their standard errors, to construct the opinion of a ‘typical’ assessor (among those included). We chose this approach in preference to the linear opinion pool or the logarithmic opinion pool, where the pooled opinion from multiple assessors represents weakened or strengthened knowledge respectively compared with a single assessor (O’Hagan *et al.*, 2006). In our approach, central rather than extreme opinions dominate the analysis. In cases of genuinely divergent opinions between assessors, we would consider it better to acknowledge this elsewhere, through discussion and presentation of bias-adjusted meta-analyses for each assessor separately, rather than to combine the opinions statistically.

It was necessary to choose a set of bias categories which may be assumed independent, since correlations between biases would be difficult to elicit and empirical evidence is unlikely to be available. The assumption is perhaps least comfortable when considering the category ‘other bias suspected’, which may be correlated with other categories of internal bias. The correlation could be positive if considerable deficiencies in a study lead us to suspect additional biases which are less easily detected from the publication, or it could be negative if so many biases are apparent that we believe that there is little scope for additional bias. Another example in the anti-D immunoglobulin studies is the external biases of dose and timing, which would have been better considered as a single bias rather than as two supposedly independent biases. By failing to acknowledge a positive correlation between two biases, we would underestimate the total bias-adjusted variance (or would overestimate it if failing to acknowledge a negative correlation). Individual biases are likely to be positively correlated across trials. Since we do not take this into account, we expect our degree of variance adjustment to be conservative, meaning that the study results should be downweighted further.

The decision to assume a direct form for the biases affecting the intervention effect was based on the view that constructing separate models for every bias would be unrealistic in most practical settings. We also chose to model biases as either additive or proportional, as a simplification which we expect to be adequate in many circumstances. Within our approach, it would be possible to incorporate more complex forms of adjustment for certain biases, by adjusting for these before the other biases. In observational studies, it may be desirable to use a detailed model of adjustment for uncontrolled confounding (Greenland, 2003; Schneeweiss *et al.*, 2005), particularly where studies are reported in more detail than in the anti-D immunoglobulin example. In randomized trials, we may wish to adjust carefully for the bias due to attrition, by including prior beliefs about informative missingness (White *et al.*, 2008).

Our chosen elicitation process was a compromise between group and individual elicitation, and this worked well from a practical point of view. It would be preferable for the team of bias assessors to include researchers from several different disciplines, as are routinely found in a team carrying out an evidence synthesis. We suggest that it is sufficient to elicit opinions from four or five assessors. Although larger numbers could improve generalizability, it is important that the individuals who are selected are knowledgeable and sufficiently motivated to take part in what may be a time-consuming and somewhat challenging process. In our experience, for each study, the bias elicitation process required an hour, plus at least an hour's preparation, and completion of the bias checklist (by one individual only) required at least three hours.

If carrying out analyses to inform politically sensitive decisions concerning availability of particular treatments, it would be important for stakeholders to approve the set of bias assessors in advance. Completed bias checklists and summaries of bias distributions that were elicited for each included study could subsequently be made publicly available. In the event of conflict over the bias-adjusted results, stakeholders could look for cases of disagreement between assessors and undertake sensitivity analyses accordingly by using software made publicly available on the Web. This process would promote transparency in the synthesis of available evidence.

The quantification of biases by assessors on elicitation scales would benefit enormously from the availability of interactive elicitation software, as recommended by O’Hagan *et al.* (2006). This would provide the opportunity for assessors to see the distributions fitted to their ranges and to modify them if they do not adequately reflect their beliefs, and to be alerted to any likely errors or inconsistencies in the ranges that are chosen. For example, assessors could be shown their chosen distribution with tertiles marked, to check that they believe the three resulting intervals to be equally probable. Similarly, it would be useful to mark extreme percentiles of the distribution. Ideally, the software would allow users to adjust the central 67% range limits, and simultaneously to update the implied distribution. Interactive software would also offer a means of showing assessors relevant empirical evidence when available.

If the aim of a meta-analysis is to summarize the literature rather than to address a target question, there is no need to adjust for external biases since differences between the (idealized) studies are accepted as diversity. The need to adjust for internal biases remains and our method can be applied directly to these.

In the anti-D immunoglobulin example, it was previously unclear how to make best use of the information that is available from studies which showed clear evidence of internal bias and which varied in relevance to the target UK setting. Our bias-adjusted meta-analysis was carried out to exemplify the methods rather than as a definitive analysis of these data. It would be preferable to involve assessors from several disciplines at the elicitation stage rather than only two (biostatistics and obstetric medicine), including at least one assessor with specialist knowledge of transfusion medicine. We place least confidence in our assessments of the external biases due to differences in dose and frequency of anti-D immunoglobulin.

Our methods for bias adjustment are presented in the context of a two-arm comparison of a binary outcome, but they could be modified for other settings. Other types of outcome could be addressed by adapting the elicitation scales and questions. For a non-comparative target setting, such as estimation of a prevalence, changes to the bias checklists would also be required.

When deciding how to handle biases in evidence synthesis, researchers should consider whether their aim is to answer a specific target question by using the evidence which is currently available, or to summarize the literature. Our methods have been developed with the first of these aims in mind. When a decision must be taken on the basis of the evidence that is available, it is essential to exploit fully the information in the studies and, for this, detailed bias assessment and elicitation are worthwhile. The element of judgement could be considered controversial, but we feel that even stronger judgements are always made, when eliminating evidence on somewhat arbitrary grounds and treating the evidence included as ‘perfect’. Our approach makes judgements explicit, transparent and open to sensitivity analysis. This can only increase the accountability of decisions that are made by organizations such as the NICE.
